# Determinants of Nutrition Facts Table Use by Chinese Consumers for Nutritional Value Comparisons

**DOI:** 10.3390/ijerph19020673

**Published:** 2022-01-07

**Authors:** Zeying Huang, Haijun Li, Jiazhang Huang

**Affiliations:** 1 Institute of Food and Nutrition Development, Ministry of Agriculture and Rural Affairs, Beijing 100081, China; huangzeying@caas.cn; 2 School of Information &Intelligence Engineering, University of Sanya, Sanya 572022, China; haijunli1968@163.com

**Keywords:** nutrition facts table, nutritional value, prepackaged food, Chi-squared automatic interaction detection, decision tree

## Abstract

The nutrition facts table is a nutrition labeling tool designed to inform consumers of food nutritional contents and enable them to make healthier choices by comparing the nutritional values of similar foods. However, its adoption level is considerably low in China. This study employed the Chi-squared Automatic Interaction Detection (CHAID) algorithm to explore the factors associated with respondents’ adoption of nutrition facts table to compare the nutritional values of similar foods. Data were gathered through a nationally representative online survey of 1500 samples. Results suggested that consumers’ comprehension of the nutrition facts table was a direct explanatory factor for its use. The usage was also indirectly explained by people’s nutrition knowledge, the usage of nutrition facts table by their relatives and friends, and their focus on a healthy diet. Therefore, to increase the use of nutrition facts table by Chinese consumers, the first consideration should be given to enhancing consumers’ comprehension of the labeling

## 1. Introduction

As a description of the nutritional properties of food [1], nutrition labeling is regarded as a critical instrument to meet consumers’ need for accurate and comprehensible nutrition information to make healthier choices [2]. The nutrition facts table is a standardized statement of the nutrient content of foods [1], and it has been applied in the United States, Britain, and Canada. As a food label in China since 2013, the nutrition facts table has mandatorily provided the information about energy value and the amounts of protein, fat, carbohydrates, and sodium, as well as the percentages of Nutrient Reference Values (NRV) per 100 g/mL on all prepackaged food items regulated by the national standard [3]. Under the circumstance that Chinese people are increasingly buying unhealthy prepackaged foods such as fatty foods and sugary drinks [4], the nutrition facts table is designed to help Chinese consumers to understand the nutritional status of food, which is expected to enable them to choose healthy food (i.e., low-calorie, low-fat, low-sodium, and high-protein) by comparing the nutritional information of food products among various brands or different series of the same brand. However, a low level (19.2%) of Chinese residents’ health literacy was found in the 2019 national health literacy monitoring results [5], and it is likely to influence people’s ability to or interest in reading food labels [6]. In practice, a nutrition facts table is rarely used in China [7], not to mention using it to compare the nutritional values. A representative survey showed that 70% of Chinese participants claimed to rarely or never use nutrition labels when shopping for food [8]. Hence, more attention should be paid to understanding the drivers of nutrition facts table use by consumers and devising appropriate measures to promote its usage.

Existing studies mainly employed a logistic regression model to identify the adoption of nutrition labeling by consumers [9,10]. However, logistic regression has inherent disadvantages in studying nutrition labeling usage, a complex human behavior influenced by a wide range of interrelating factors because it cannot reflect various characteristics of such a complex practice. As a heuristic decision tree modeling method, the Chi-squared Automatic Interaction Detector (CHAID) algorithm proposed by Kass is an alternative and more efficient approach to logistic regression models as it classifies key determinants quickly and effectively by developing a decision tree [11]. In recent years, the CHAID algorithm is widely used in the medical field [12,13], yet few applications have been conducted on nutrition labeling. To the best of our knowledge, the present study is the first in China to apply the comprehensive CHAID model to examine nutrition labeling use. The findings are expected to contribute to providing a scientific basis and theoretical reference for increasing the use of nutrition labeling in China and other countries.

## 2. Methods and Materials

### 2.1. Methods

CHAID algorithm which starts with the full data set is implemented by repeatedly splitting subsets of the space (i.e., a mathematical concept where one set belongs to another) into two or more child nodes (i.e., a node that has a node attached to itself rather than the root node) [14]. It is an operation standard procedure to conduct a literature review to identify potential factors that affect human behavior before the application of the CHAID algorithm, and the factors mentioned which are excluded in the dataset could be ignored [15]. Prior to CHAID decision tree analysis, potential factors affecting consumers’ behavior of nutrition labeling usage were obtained from a literature review (see Table 1) and then the Chi-square test was used to assess whether the difference was statistically significant between respondents’ characteristics and label use. All characteristics whose *p*-value of Chi-square statistic was statistically significant (*p*-value < 0.05) were included as independent variables in decision tree models.

To determine the most favorable split at any node, the most significant variable is firstly selected using a Chi-squared independence test. Next, using chi-squared statistics, the optimal points for dividing the selected variable are calculated until they could not be divided, resulting in the amount of optimal points. A database including the entire input variable is divided into sub-databases along the optimal points. This ends one loop in the algorithm for the decision tree. The same process is carried out in each sub-database. Tree growth is stopped when there are no significantly different chi-squared statistics [16]. The parameters for conducting the algorithm don’t have to be determined due to nonparametric estimation [17]. The following three parameters are set in this study to conduct CHAID algorithm: (1) *α* for splitting = 0.05, which specifies the significance level for splitting nodes; (2) *α* for merging = 0.05, which specifies the significance level for merging categories; and (3) the maximum number of iterations = 100, which specifies the maximum number of iterations before stopping. The Chi-square test and the CHAID decision tree were conducted using SPSS version 19.0.

### 2.2. Data Collection Data

We used the variables from the literature review (see Table 1) to design a self-administered questionnaire that contained 21 questions including socio-demographic information (see Supplementary Materials for details: Table S1). The Wenjuanxing platform (https://www.wjx.cn accessed on 29 July 2020) is one of the leading companies specializing in online questionnaire data collection in China and has a sample pool of 2.6 million potential respondents reasonably distributed by gender, age, occupation, and region. Paid survey data collection services are provided by the Wenjuanxing platform for sending questionnaires to target samples and ensuring the validity of questionnaire information.

With the development of the transportation and food industry, various types and quantities of prepackaged food across the country are in sufficient supply [32]. Generally, most of the Chinese people choose their favorite food by comparison. From 29 July to 21 August 2020, the Wenjuanxing platform was commissioned to collect 1500 valid survey samples nationwide from the sample pool. First of all, the platform employed a stratified sampling approach to select randomly 54 individuals from each of China’s 30 provinces/autonomous regions (except Tibet) to finish online surveys in simplified Chinese suitable for various regions and participants received an incentive with 8 yuan RMB. Then a double check was conducted among the collected 1620 questionnaires to eliminate invalid ones due to lack of information and implausible answers. Finally, 1500 valid samples totally were generated for analysis.

## 3. Results

### 3.1. Sample Characteristics

Our sample is representative of the Chinese population in terms of socio-demographic characteristics. Table 1 describes the socio-demographic features of the sampled 1500 individuals. Overall, the most probable profile of a respondent is male (58.07%), aged between 18 and 44 years (66.80%), had a middle-income between 10,000 and 50,000 Yuan after tax (21.27%), and attended a junior high school (42.40%). The nutrition facts table was far from being effectively adopted and only less than 40% reported that they compared the nutritional value of similar foods using the labeling at the point of purchase. 

### 3.2. Chi-Square Test Result

As can be seen from Table 2, the independent variables with *p*-value < 0.05 were identified as statistically significant determinants, including the respondent’s education, BMI, health self-rating, nutrition knowledge level, comprehension of nutrition facts table, and whether one focuses on an individual healthy diet, limits food consumption to prevent obesity, considers nutrition facts table helpful in healthy food choice, and has friends and relatives using nutrition facts table.

### 3.3. CHAID Algorithm Analysis

Based on the Chi-square test result, the CHAID algorithm was performed by using a comparison of the nutritional value as the dependent variable. The characteristics of the initial model were shown in Table 3, the maximum tree depth was 3, and there were 12 nodes and 7 terminal nodes in total. The minimum cases in the parent node and child node were 119 and 8, respectively. As for the outcomes, there were only 4 independent variables (i.e., respondents’ nutrition knowledge level, whether to focus on an individual healthy diet, comprehension of nutrition facts table, and whether friends and relatives use nutrition facts table) selected by the CHAID algorithm.

Table 4 illustrates the Chi-square test results of terminal nodes. All independent variables were selected by the CHAID algorithm. For instance, the comprehension of the nutrition facts table were grouped into 3 categories, and nutrition knowledge level was divided into 2 categories. All terminal nodes with *a p*-value < 0.05 were statistically significant, suggesting that the CHAID algorithm was reasonable.

**Figure 1:**
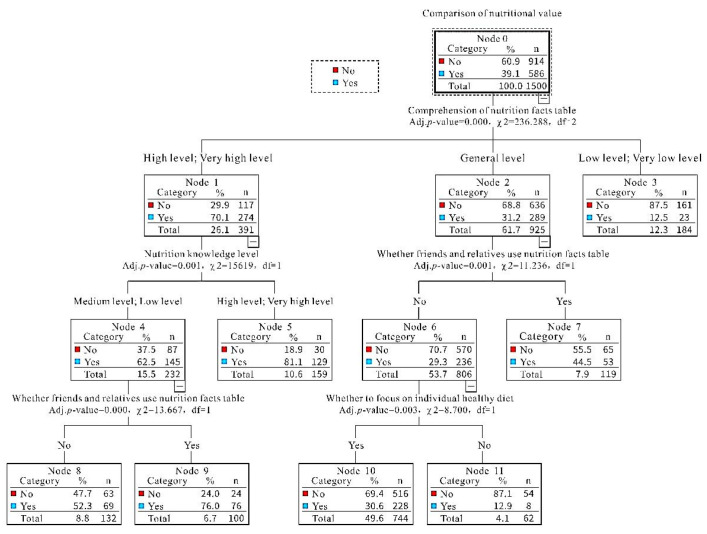
Results of Decision Tree Analysis based CHAID algorithm. Note: n (number); df (degree of freedom); χ^2^ (chi-square); Adj. *p*-value (adjusted *p*-value).

The CHAID decision tree diagram for comparison of nutritional value was indicated in Figure 1. The decision tree was firstly branched based on respondents’ comprehension of the nutrition facts table and then branched according to their nutrition knowledge level, whether to focus on an individual healthy diet, and whether friends and relatives use the nutrition facts table. Low AUC (Area under Curve) of the CHAID algorithm was 0.732 (*p* < 0.000), which was more than 0.5, and suggested that it was suitable to explore factors influencing the comparison of nutritional values via the CHAID algorithm. 

The direct and strongest associated factor was respondents’ comprehension of the nutrition facts table. People with a very high or high level of comprehension of the nutrition facts table (70.1%) were most likely to compare the nutritional values of similar foods. 

It was also found that respondents’ nutrition knowledge level was the main determinant among those with a very high or high level of comprehension of nutrition facts table, and those with a very high or high level of nutrition knowledge (81.1%) showed a greater propensity to adopt this practice. Whether friends and relatives use nutrition facts table was the main determinant amongst a sample of those with a medium level of comprehension of the label and medium or low level of nutrition knowledge, and respondents whose friends and relatives used nutrition facts table were most likely to compare nutritional values of similar foods. Moreover, whether to focus on an individual healthy diet was the main determinant among those without friends and relatives using the nutrition facts table, and respondents who focused on an individual healthy diet (30.6%) were most likely to compare nutritional values of similar foods.

## 4. Discussion

This study provided new insights from an innovative method for exploring factors affecting nutrition labeling use. CHAID algorithm was proved to be a suitable model to fit the survey data. Also, the graph structure generated by the CHAID tree is highly visual, which makes the interaction between variables intuitively understood. 

As noted, the only direct factor was consumers’ comprehension of the nutrition facts table, which largely determined the adoption behavior. It was in line with the findings of Hobin et al. [28]. Specifically, the higher the respondents’ comprehension was, the more likely they were to compare nutritional values. This is because consumers found it hard to compare nutritional values of similar foods by using the information of nutrients’ contents and their NRV% on the nutrition facts table if they knew little about the concepts and functions of carbohydrates, fat, protein, sodium, and NRV%. 

Respondents’ nutrition knowledge level, whether friends and relatives use nutrition facts table, and whether to focus on individual healthy diet played an indirect role during their decision process of adoption. First, respondents’ nutrition knowledge level was positively correlated with their behavior of comparing the nutritional value of similar foods. The higher the respondents’ nutrition knowledge level was, the more scientific understanding of health-promoting eating styles they had. It could assist consumers in increasing the use of labeling. Second, whether friends and relatives use the nutrition facts table was found to positively impact the adoption behavior of those with an average level of comprehension or a low and medium level of nutrition knowledge. In reality, people in China often go food shopping with friends/relatives [33]. Respondents might be encouraged to compare nutritional values of similar foods by using the nutrition facts table once their friends and relatives used the labeling when food shopping. Even though the respondents had medium-level comprehension of the nutrition facts table or a low level of nutrition knowledge, they could be encouraged to compare nutritional values of friends and relatives around used nutrition labeling. Third, whether to focus on an individual healthy diet was positively related to food label use of respondents who had an average level of comprehension of the nutrition facts table and had no friends or relatives using the nutrition facts table. If an individual paid attention to a healthy diet in daily life, he or she might know how to maintain and improve health. Even though respondents who generally focused on the healthy diet had an average level of understanding of nutrition facts table information or no friends or relatives used nutrition labeling, he or she would compare the labeling information of similar foods, particularly the nutrient contents and NRV%, at the point of purchase.

Additionally, four variables including individual education level, self-ratings of health, whether to have limited foods to prevent obesity, whether the nutrition facts table is helpful in healthy food choice, were not selected by the CHAID algorithm due to little effects on the nutrition facts table use for nutritional values comparison. There were some possible reasons. Firstly, those with high education levels were found no association with a high knowledge level of food labeling [34], and personal subjective evaluation of health status could hardly reflect the requirement and practice of their own health promotion such as food labels usage [35]. Secondly, people who choose diets for body weight control and health follow advice from professionals in most cases rather than nutrition information in labels [36]. Thirdly, individuals who could trust nutrition labels rarely understood how to use them if they had a low level of relevant knowledge [37].

However, several limitations need to be noted. First, although the CHAID tree demonstrates clearer and more available classification information, it fails to reflect the main effect and superposition effect of variables [38]. Thus, to obtain better results, future studies should select the main effect of statistically significant variables by logistic regression, and subsequently analyze the interaction between variables by a decision tree model. Second, the survey questions design needs to be improved. On one hand, both some questions which contained double-barreled items and the response options only with "yes" and "no" for the item on friends/relatives’ use of the nutrition facts table were less likely to reflect accurate responses, so it is necessary to make items clear in the next step of questionnaire design to avoid double-barreled questions and inadequate options. On the other hand, survey respondents probably had different responses to the practice of nutrition facts table due to food purchase for the unclear target population, resulting in inaccurate data collection. Third, although self-administered questionnaires could collect data efficiently, participants may still make inaccurate answers or fail to complete unclear questions in the online survey environment. This perhaps leads to poor data quality. Complex indirect questioning methods, such as the list experiment, are expected to overcome the difficulties facing high-quality data collection. Finally, we failed to observe any demographic differences or differences in 50 surveyed consumers’ use of the nutrition facts table by region. There may be important differences that have implications for addressing consumer behavior with unique approaches in different regions. These would be dealt with in the future study.

## 5. Conclusions

The current research aimed to explore the key determinants of Chinese consumers’ nutrition facts table use for nutritional values comparison among similar foods. The findings revealed that the CHAID algorithm could identify factors scientifically and effectively, which proved to be a well-suited identification method. Consumers’ comprehension of the nutrition facts table was the direct motivator that could largely explain label use. Besides, respondents’ nutrition knowledge, whether relatives and friends use the nutrition facts table, and whether to focus on a healthy diet were indirect factors. 

## 6. Recommendations

To encourage consumers to develop a good habit of using the nutrition facts table to compare nutritional values of foods, the following policy recommendations are offered. (1) Nutrition facts table in China showed nutrition information in terms of professional terms, words, and values in a single and abstract manner, which is not easy to understand. To improve consumers’ understanding of the nutrition facts table, a variety of popular forms should be conducted to promote the nutrition facts table, especially the concepts and functions of energy, key nutrients, and NRV%. (2) Nutrition knowledge learning should not only become a key course for primary and middle school students but also be promoted to the community by nutritionists. (3) Some important communities, schools, and companies should be encouraged to pilot the use of nutrition labeling, creating a sound social atmosphere that is conducive to the adoption of nutrition facts table in the wider population. (4) Despite an increasing number of consumers who pay attention to a healthy diet, many Chinese consumers still have a low level of awareness, resulting in unhealthy patterns of food consumption and nutrition intake, which highlights the need to strengthen education programs that aim at guiding public attention towards a balanced diet and nutrition intake.

## Figures and Tables

**Table 1:** Variables initially identified through a literature review.

Variables	Definition	Samples	Percentage%	References
Comparison of nutritional value	No	914	60.93	Webb [18]
	Yes	586	39.07	
Gender	Male	629	41.93	Gupta & Dharni [19]
	Female	817	58.07	
Age	17 years old or below	300	20.00	Govindasamy & Italia [20]
	18–44 years old	1002	66.80	
	45–59 years old	189	12.60	
	60 years old or above	9	0.60	
Marriage	Unmarried	693	46.20	McLean-Meyinsse [21]
	Married	807	53.80	
Education	Primary school or below	3	0.20	Krešić & Mrduljaš [22]
	Junior school	36	42.40	
	Senior school	373	34.87	
	Junior college or undergraduate	992	18.13	
	Postgraduate or above	96	4.40	
BMI ^a^	Underweight (<18.5)	275	18.33	Department of Disease Control, Ministry of Health, PRC [23]
	Normal (18.5–23.9)	939	62.60	
	Overweight (24–28)	211	14.07	
	Obese (>28)	75	0.05	
Annual household incomeafter-tax (Yuan) ^b^	<10,000	127	8.47	McLean-Meyinsse [21]
	10,000–49,999	319	21.27	
	50,000–99,999	315	21.00	
	100,000–149,999	299	19.92	
	150,000–199,999	226	15.07	
	≥200,000	214	14.27	
Live in urban areas	No	450	30	Govindasamy & Italia [20]
	Yes	1050	70	
Health self-rating	Very poor	5	0.34	Zhang et al., [24]
	Poor	23	1.53	
	General	317	21.13	
	Good	772	51.47	
	Very good	383	25.53	
Nutrition knowledge level ^c^	Low	106	7.07	Christoph et al., [25]
	Medium	970	64.67	
	High	397	26.47	
	Very high	27	1.80	
Whether to focus on an individual healthy diet	No	124	8.27	Cooke & Papadak [26]
	Yes	1376	91.73	
Whether to have limited foods to prevent obesity	No	1069	71.27	Frieden et al., [27]
	Yes	431	28.73	
Comprehension of nutrition facts table	Very low	40	2.67	Hobin et al., [28]
	Low	144	9.60	
	General	925	61.67	
	High	314	20.93	
	Very high	77	5.13	
Whether nutrition facts table is helpful in healthy food choice	No	66	4.40	Sun et al., [29]
	Yes	1434	95.60	
Whether friends and relatives use the nutrition facts table	No	1194	79.60	Rose et al., [30]
	Yes	306	20.40	

Note: ^a^ BMI stands for Body Mass Index which is a ratio of a person’s weight to their height; ^b^ One US dollar is equal to 6.941 Chinese Yuan and One Euro is equal to 8.199 Chinese Yuan from 29 July to 21 August 2020. ^c^ Each respondent’s nutrition knowledge level was evaluated by the six declarative knowledge questions from Dietary Guidelines for Chinese Residents [31] (see Supplementary Materials for details). The correct answer proportions of 0–25%, 26–50%, 51–75%, and 76–100% indicate low level, medium level, high level, and very high level, respectively.

**Table 2:** Chi-square test results of subgroups from the comparison of nutritional value.

	Yes = 1 [*n* (%)]	No = 0 [*n* (%)]	Chi-Square	*p*-Value
Male (*n* = 629)	237 (37.68)	392 (62.32)	0.876	0.349
Female (*n* = 871)	349 (40.07)	522 (59.93)		
≤17 years old (*n* = 300)	226 (75.33)	74 (24.67)	5.842	0.120
18–44 years old (*n* = 1002)	280 (27.94)	722 (72.06)		
45–59 years old (*n* = 189)	79 (41.80)	110 (58.20)		
≥60 years old (*n* = 9)	1 (11.11)	8 (88.89)		
Unmarried (*n* = 693)	257 (37.09)	436 (62.91)	2.125	0.145
Married (*n* = 807)	329 (40.77)	478 (59.23)		
Primary school or below (*n* = 3)	0 (0)	3 (100)	15.081	0.005
Junior school (*n* = 36)	5 (13.89)	31 (86.11)		
Senior school (*n* = 373)	31 (8.31)	342 (91.69)		
Junior college or undergraduate (*n* = 992)	527 (53.12)	465 (46.88)		
Postgraduate or above (*n* = 96)	23 (23.96)	73 (76.04)		
Underweight (*n* = 275)	93 (33.82)	182 (66.18)	8.176	0.043
Normal (*n* = 939)	392 (41.75)	547 (58.25)		
Overweight (*n* = 211)	77 (36.49)	134 (63.51)		
Obese (*n* = 75)	24 (32.00)	51 (68.00)		
Annual household income after tax				
<10,000 Yuan (*n* = 127)	29 (22.83)	98 (77.17)	6.389	0.270
10,000–49,999 Yuan (*n* = 319)	92 (28.84)	227 (71.16)		
50,000–99,999 Yuan (*n* = 315)	109 (34.60)	206 (65.40)		
100,000–149,999 Yuan (*n* = 299)	117 (39.13)	182 (60.87)		
150,000–199,999 Yuan (*n* = 226)	147 (65.04)	79 (34.96)		
≥200,000 Yuan (*n* = 214)	92 (42.99)	122 (57.01)		
Live in rural areas (*n* = 450)	170 (20.44)	280 (79.56)	0.449	0.503
Live in urban areas (*n* = 1050)	416 (28.44)	634 (71.56)		
Health self-rating				
Very poor (*n* = 5)	1 (20.00)	4 (80.00)	38.780	0.000
Poor (*n* = 23)	4 (17.39)	19 (82.61)		
Average (*n* = 317)	93 (29.34)	224 (70.66)		
Good (*n* = 772)	295 (38.21)	477 (61.79)		
Very good (*n* = 383)	193 (50.39)	190 (49.61)		
Nutrition knowledge level				
Low (*n* = 106)	31 (29.25)	75 (70.75)	64.342	0.000
Medium (*n* = 970)	324 (33.40)	646 (66.60)		
High (*n* = 397)	211 (53.15)	186 (46.85)		
Very high (*n* = 27)	20 (74.07)	7 (25.93)		
Whether to focus on an individual healthy diet				
No (*n* = 124)	103 (83.06)	21 (16.94)	27.813	0.000
Yes (*n* = 1376)	811 (58.94)	565 (41.06)		
Whether to have limited foods to prevent obesity				
No (*n* = 1069)	696 (65.11)	373 (34.89)	27.232	0.000
Yes (*n* = 431)	218 (50.58)	213 (49.42)		
Comprehension of nutrition facts table				
Very low (*n* = 40)	34 (85.00)	6 (15.00)	238.093	0.000
Low (*n* = 144)	127 (88.19)	17 (11.81)		
Average (*n* = 925)	636 (68.76)	289 (31.24)		
High (*n* = 314)	89 (28.34)	225 (71.66)		
Very high (*n* = 77)	28 (36.36)	49 (63.64)		
Whether nutrition facts table is helpful in healthy food choice				
No (*n* = 66)	51 (77.27)	15 (22.73)	7.743	0.005
Yes (*n* = 1434)	863 (60.18)	571 (39.82)		
Whether friends and relatives usenutrition facts table				
No (*n* = 1194)	804 (67.34)	390 (32.66)	100.816	0.000
Yes (*n* = 306)	110 (35.95)	196 (64.05)		

Source: Authors’ own calculations.

**Table 3:** Characteristics of the initial model and its outcomes.

Model	Model Attributes	Details
Initial model before CHAID algorithm use	Independent variables to be selected	Education
		BMI
		Health self-rating
		Nutrition knowledge level
		Whether to focus on an individual healthy diet
		Whether to have limited foods to prevent obesity
		Comprehension of nutrition facts table
		Whether nutrition facts table is helpful in healthy food choice
		Whether friends and relatives use nutrition facts table
	Maximum tree depth	3
	Minimum cases in the parent node	119
	Minimum cases in the child node	8
Outcomes	Independent variables selected	Nutrition knowledge level Whether to focus on an individual healthy diet Comprehension of nutrition facts table Whether friends and relatives use the nutrition facts table
	Number of nodes	12
	Number of terminal nodes	7
	Depth	3

Source: Authors’ own calculations. Note: the maximum tree depth is the amount of growth layers of a decision tree; the terminal node is the node that can no longer be divided.

**Table 4:** Chi-square test results of terminal nodes [*n* (%)].

Terminal Nodes	Comparison of Nutritional Value			Chi-Square	*p*-Value
	Yes	No	Total		
Very low or low level of comprehension of nutrition facts table	23 (1.53)	161 (10.73)	184	236.288	0.000
Friends and relatives use the nutrition facts table	53 (3.53)	66 (4.40)	119	11.236	0.001
Focus on an individual healthy diet	228 (15.20)	516 (34.40)	744	8.700	0.003
Not focus on an individual healthy diet	8 (0.53)	54 (3.60)	62	8.700	0.003
Very high or high level of nutrition knowledge	129 (8.60)	30 (2.00)	159	15.619	0.001
Friends and relatives use the nutrition facts table	76 (5.07)	24 (1.60)	100	13.667	0.000
Friends and relatives do not use the nutrition facts table	69 (4.60)	63 (4.20)	132	13.667	0.000

Source: Authors’ own calculations.

## Data Availability

Data is contained within the article.

## Supplementary Materials

The following supporting information can be downloaded at: https://www.mdpi.com/article/10.3390/ijerph19020673/s1, Table S1: The questionnaire.
