# Supplementary material for: Determinants of Nutrition Facts Table Use by Chinese Consumers for Nutritional Value Comparisons
Source: Int J Environ Res Public Health. 2022 Jan 7;19(2):673. doi: 10.3390/ijerph19020673 (PMC8775507; doi:10.3390/ijerph19020673)
Supplement: Supplementary file 1 [file ijerph-19-00673-s001.zip › ijerph-1498965-supplementary.pdf]

Supplementary Materials

Table S1: The questionnaire

Section 1 Cognition and use of nutrition facts table

Q1-1 Do you compare nutritional value of similar foods by using nutrition facts table at the point of purchase? A.Yes B. No

Q1-2 What do you know about the information on the nutrition facts table?

A. Very little B. Little C. General D. much E. Very much

Q1-3 Do you think it is helpful in healthy food choice by using nutrition facts table?

A.Yes B. No

Q1-4 Do your friends and relatives use nutrition facts table? A.Yes B. No

Section 2 measurement of nutrition knowledge level:

Q2-1 I know that the diet should be varied and grain-based. A. Yes B. No

Q2-2 I know how to have a balanced diet and maintain a healthy weight. A. Yes B. No

Q2-3 I know how to have more fruits, vegetables, dairy and soy. A. Yes B. No

Q2-4 I know how to have fish, poultry, eggs and lean meat in moderation. A. Yes B. No

Q2-5 I know how to have less salt, oil, sugar and alcohol. A. Yes B. No

Q2-6 I know how to eliminate waste and try new things. A. Yes B. No

Section 3 Individual dietary

Q3-1 Do you focus on individual healthy diet? A. Yes B. No

Q3-2 Are you in the habit of eating only limited foods to prevent obesity? A. Yes B. No

Section 4 Demographic characteristics

Q4-1 Your gender : A. Male B. Female

Q4-2 Your age: A. under 18 years old B. 18-44 years old C.45-59 years old D. 60 years old and above

Q4-3 Your height:______ cm

Q4-4 Your weight:______ kg

Q4-5 Your marriage: A. married B. unmarried or divorce

Q4-6 Your education level: A. Primary school or below B. Junior school C. Senior school D. Junior college or undergraduate E. Postgraduate or above

Q4-7 Your annual household income after tax (Yuan):

A.<10,000 B. 10,000–49,999 C. 50,000–99,999 D. 100,000–149,999 E. 150,000–199,999 F.≥200,00

Q4-8 You live in urban areas: A. Yes B. No

Q4-9 How do you rate your general health? A. Very poor B. Poor C. General D. Fine E. Very well
